# Optimizing the community resource specialist to address social needs in primary care: results from a pragmatic quality improvement evaluation

**DOI:** 10.1186/s12875-025-02922-x

**Published:** 2025-10-31

**Authors:** Cara C. Lewis, Michael D. Pullmann, Clarissa Hsu, Consuelo Norris, Jessica Mogk, Roy Pardee, Callie Walsh-Bailey, Emily Westbrook, Amy Lee, Jessica Ridpath, Cindee DeWitt, Ammarah Mahmud, Katie Coleman, Paula Lozano

**Affiliations:** 1 Kaiser Permanente Washington Health Research Institute, 1730 Minor Avenue, Suite 1600, Seattle, WA 98101 USA; 2 Seattle, WA USA; 3 Department of Medical Social Sciences, Northwestern University Feinberg School of Medicine, 625 N. Michigan Av, Chicago, IL 60611 USA; 4 Kaiser Permanente Washington Health Plan, 2715 Naches Ave. SW, Renton, WA 98057 USA; 5 Research to Practice LLC, Seattle, Washington USA

**Keywords:** Social health, Social risk, Social needs, Primary care, Quality improvement, Implementation, Practice facilitation

## Abstract

**Background:**

Social care integration in health systems is on the rise in the United States, particularly since the National Committee for Quality Assurance introduced screening and intervention as HEDIS metrics. These policy levers outpace empirical knowledge to guide how best to operationalize social care. This study reports results from a quality improvement initiative to implement social care in an integrated health system.

**Methods:**

A quantitative effectiveness evaluation was conducted across 32 clinics in Kaiser Permanente Washington, which had recently embedded Community Resource Specialists (CRS) in their primary care teams and integrated a social health screener into their electronic health record. Using a pragmatic design with propensity score matched comparison group (PSC), we compared two intervention arms (both of whom completed a social health screener): (1) CRS-S who engaged in only a single CRS visit and (2) CRS-M who engaged in at least two CRS visits. Patients completed a survey shortly after their qualifying primary care encounter and approximately three months later that assessed the following domains: social health, patient experience with the care team, and health and functioning; healthcare utilization was obtained from the electronic health record. Patients from each arm were then purposefully sampled for qualitative interviews.

**Results:**

Quantitative results suggest that CRS-M patients experienced exacerbated social risk severity and food insecurity over three months, but improved financial risk. For the majority of domains, no differences were observed between arms, though CRS-M demonstrated poorer coping over time whereas PSC patients showed higher use of instrumental and emotional support coping strategies. CRS-M reported worse health and need for more help with activities of daily living, but improvements in trust in their care team. Qualitative results showcased, by design, the positive potential impact of working with a CRS across all domains of interest, especially social and mental health.

**Conclusion:**

This quality improvement evaluation of social care integration using the CRS illustrates a potential pathway for activating social support and healthcare relationships in primary care, but more rigorous designs and longer-term follow up are needed to explore if this pathway leads to improvements in patient or population health and healthcare utilization.

**Supplementary Information:**

The online version contains supplementary material available at 10.1186/s12875-025-02922-x.

## Introduction

*Social determinants of health *are upstream factors (e.g., structural racism, neighborhood walkability, poverty) that have a profound impact on one’s health, over and above the effects of medical intervention and genetics [1–3]. These structural, political, and societal issues manifest in *social risks*, such as housing and food insecurity, financial strain, and social isolation for individuals and their families. Not everyone experiencing social risk desires help, but a significant proportion endorse *social needs *for which they would like health system support [4, 5].

A 2017 systematic review of *social health* interventions (i.e., those addressing social risk and/or needs) delivered by health systems yielded equivocal findings in terms of physical health, utilization, and cost outcomes [6]. More consistently, these types of interventions appear to positively impact factors related to patient experience (e.g., trust in the care team) [7], attendance and engagement [8, 9], and amelioration of social needs [6, 10]. Despite this variability in findings, recent National Committee for Quality Assurance HEDIS metrics have created new incentives for health systems to screen for social risk and address social needs, often with interventions that support information sharing and linkages to community-based resources [11].

To proactively address social needs of patients, Kaiser Permanente Washington (KPWA) partnered with its embedded research institute (KPWHRI), patients and providers to codesign a lay health worker role—the Community Resource Specialist (CRS), which was piloted in three primary care clinics [12] (PCORI CER1011) [13]. Similar to Community Health Workers [14], though specialized around social needs and embedded in the primary care setting, the CRS role focused on three key interrelated activities: 1) assisting patients to access community resources and set health-related goals using motivational interviewing, 2) developing relationships with local resource providers, and 3) increasing the primary care team’s knowledge of patient social needs and available resources. Patient experience and care team satisfaction results from the pilot study were so promising (e.g., 86% of patients made progress on their goals; care team members reported an ease in their workload) that KPWA invested in the scale and spread of the CRS role to its 32 primary care clinics.

To optimize CRS integration, KPWA again partnered with KPWHRI through its internally funded learning health system (LHS) program [15, 16] which provided implementation support and conducted an evaluation. The implementation support included practice facilitation (PF) in three diverse clinics, electronic health record (EHR) enhancements to enable embedded social health screening and patient tracking, and training in motivational interviewing for all CRSs. Simultaneously, the LHS team conducted a pragmatic quality improvement evaluation focused on three key domains that serve as an early test of SIREN’s social care logic model [17]. We hypothesized that CRS encounters would result in improved: (1) patient social needs and self-efficacy; (2) patient coping, symptoms, and functioning; and (3) patient engagement and utilization of health care services.

## Methods

### KPWA, LHS program, and CRS integration

KPWA is an integrated health system and insurance plan with 32 primary care clinics spanning nearly 330 miles across Washington state. Clinics range in size from 7,000 to 40,000 patients. KPWA patients are relatively diverse, reflecting the racial demographics of surrounding communities. The internally funded LHS Program brings research and health system leaders together to design, implement, and evaluate programs that directly benefit members and care teams. Core capabilities of the LHS Program leveraged in the CRS optimization partnership included: use of research evidence to inform best practices for CRS integration into primary care, data and analytics in the implementation of change via PF, and rigorous measures and methods for evaluation. The KPWHRI IRB determined this quality improvement evaluation was not research (IRB#1,223,879–1). Patients could opt out of the evaluation at any time and involvement in the evaluation in no way influenced their care at KPWA.

Briefly, implementation support began with documenting the core competencies of the CRS role; developing an implementation plan to support CRS integration within the team, clinic, and organization; and an implementation and integration assessment tool to inform PF. Three of the 32 clinics engaged in PF over 12 months to surface necessary enhancements to the EHR and recommend practices for optimizing CRS integration. These clinics were chosen for their diversity in size, location, and CRS staffing. Practice facilitators collaborated with each clinic’s interdisciplinary implementation team (e.g., clinic leadership, provider, medical assistant, front desk representative, social worker, CRS, and CRS/mental health leadership) to address local barriers, enhance workflows, systematically document recommended practices that supported CRS integration, and refine the implementation plan to guide implementation at the remaining sites. Care delivery information technology was leveraged at two timepoints: (1) initially to build a social health screener into KPWA’s Epic and (2) after the second clinic completed PF to address barriers related to care team referral of patients to the CRS, and patient tracking and follow up by the CRS.

A CRS was embedded at each primary care clinic to aid relationship building with patients, the care team, and the broader community; FTE varied based on the patient census at each clinic. The CRSs were encouraged to use the Your Current Life Situation (YCLS) [18–20] social needs screening tool when initiating social care with all patients. The YCLS was incorporated into the EHR, however at the time of this evaluation, KPWA did not have the resources to conduct universal social health screening. Given this, patients were referred to the CRS by their primary care team based on care team member elicitation/knowledge of a patient’s social risks and/or needs or patient self-report or self-referral.

### Design

We launched a system-wide quantitative effectiveness evaluation after the initial round of EHR enhancements and completion of PF at the first clinic. Given the nature of this quality improvement endeavor, the design was pragmatic and included a propensity score comparison (PSC) or “counterfactual” group (see Supplementary file 1 for details) to evaluate the differential impact of single visit (CRS-S) versus multiple (CRS-M) visits with the CRS. The quantitative evaluation included a robust original data collection approach described below. In-depth qualitative interviews were conducted with the three groups of patients. The study used a sequential QUAN --> qual mixed methods approach [21] with the goal of using qualitative data to expand on quantitative findings.

### Quantitative evaluation participants

Patients with a CRS encounter were excluded from the evaluation if they had dementia, if their visit was only to complete a medical financial assistance form, or if they were still actively working with the CRS (indicated by having met with the CRS within the last 60 days). Patients were eligible for the evaluation if they spoke English or Spanish and were 18 years or older. Patients were included in the CRS-S group if they had only 1 encounter with the CRS via phone or in person in the 90-day evaluation window, or they had > 1 encounter but no YCLS documentation in their medical record. Patients were included in the CRS-M if they had at least two CRS encounters on the phone or in person that lasted longer than 5 min and they had a completed YCLS. Patient recruitment was done on a weekly rolling basis, with the goal of meeting pre-specified per-clinic, per-week target numbers for both CRS groups and PSC. When a given clinic’s target was satisfied, recruitment from that clinic was deemed complete.

Patients were eligible for the PSC group if they had never had a CRS encounter, had a telephone encounter with a non-CRS provider at one of our clinics of interest on a day when a CRS was working at that clinic, spoke English or Spanish, had an Adjusted Clinical Groups Resource Utilization Band score < 5, did not currently suffer from dementia, and had a propensity score above an assessed threshold; see Supplementary file 1 for details outlining the three-stage PSC approach. Briefly, the propensity scoring algorithm was developed in a pilot phase, with the goal of predicting patients with CRS eligibility (i.e., social needs). Patients in each group, from each clinic, were shared with KPWHRI’s survey department for telephone recruitment into the evaluation consisting of two screening questions: (1) “How easy or difficult is it for you to follow through with your care plan?” (2) “Which, if any, of the following resources would you like to receive help with at this time?” Patients who responded that it was “difficult” or “very difficult”, and/or patients who endorsed desiring help with at least one of the 13 resources (e.g. food, housing, transportation) were considered eligible for CRS services and included as PSC patients in the baseline and follow-up surveys.

### Survey design and administration

The evaluation team, in collaboration with delivery system partners, designed a survey for patient data collection informed by the PCORI-funded CRS evaluation and the extant literature that ultimately maps onto the SIREN logic model [17] (see Supplementary files 2 and 3). Previously validated measures or validated subscales from established measures were used where possible. The study team generated items to assess constructs for which there were no available measures. Two trained research specialists conducted cognitive testing with 12 representative patients to refine the survey. The final survey contained 52 items; detailed description of the survey items are available in Table 1.

**Table 1:** Patient survey domains and source measures

Domain	Construct	Measure Source	# of items	Measure Description
Primary Outcome	Self-efficacy	General Self-efficacy scale (GSE-6) [22]	6	Psychometric information: demonstrated good internal consistency and acceptable discriminant and predictive validity [22] Example item: it is easy to stick to my aims and accomplish my goals Response scale: 1 (not at all true) to 4 (exactly true) Scoring & interpretation: item scores summed; higher score indicates greater self-efficacy
Primary Outcome	Social Needs	Your Current Life Situation (YCLS) [18–20]	10	Psychometric information: Example Item: In the past 3 months, did you have trouble paying for any of the following? Example item response scale (responses vary by item): food; housing; heat and electricity; medical needs; transportation; childcare; debts; other; none of these Scoring & interpretation: selection of any response other than “none of these” indicates presence of social need
Primary Outcome	Perceived Burden of Social Needs	Your Current Life Situation (YCLS) [18–20]	1	Psychometric information: Example Item: On a scale ranging from 0 (not at all a problem) to 10 (very much a problem), how much of a problem is this for you? Response scale: 0 (not at all a problem) to 10 (very much a problem) Scoring & interpretation: higher number indicated greater perceived burden
Primary Outcome	Difficulty with Care Plan	Team generated	1	Psychometric information: NA Example Item: How easy or difficult is it for you to follow through with your care plan? Example item response scale: very easy, somewhat easy, somewhat difficult, very difficult Scoring & interpretation: higher number indicated greater difficulty
Patient Experience with Care Team	Trust in Care Team	SONNET survey	1	Psychometric information: NA Item: How much do you trust your health care providers at Kaiser Permanente? Response scale: 1 (not at all true) to 10 (completely true) Scoring & interpretation: higher score indicates greater trust
Patient Experience with Care Team	Alliance	Working Alliance Inventory (WAI-S) [23]	12	Psychometric information: has strong internal consistency [23] Example item: We agree on what is important for me to work on Response scale: 1 (strongly disagree) to 5 (strongly agree) Scoring & interpretation: goal, task, and bond alliance subscales summed separately; higher scores on each subscale indicates greater alliance in that domain
Patient Experience with Care Team	Value of time spent with CRS	Team generated item	3	Psychometric information: Example Item: Meeting with the Community Resource Specialist was worth my time Response scale: 1 (strongly disagree) to 5 (strongly agree) Scoring & interpretation: total score summed on two items; higher score indicates greater perceived value
Patient Experience with Care Team	Value of CRS Support	Team generated item	1	Psychometric information: Item: I feel supported by the Community Resource Specialist Response scale: 1 (strongly disagree) to 5 (strongly agree) Scoring & interpretation: higher score indicates greater perceived value
Health and Functioning	Depression	Patient Health Questionnaire (PHQ-2) [24]	2	Psychometric information: Example Item: Over the past 2 weeks, how often have you been bothered by any of the following problems*?* Little interest or pleasure in doing things? And Feeling down, depressed, or hopeless? Response scale: 0 (not at all) to 3 (nearly every day) Scoring & interpretation: responses to the items are summed; possible scores range from 0–6; a score greater than 3 indicates likelihood of depressive disorder
Health and Functioning	Anxiety	Generalized Anxiety Disorder (GAD-2) [25]	2	Psychometric information: Example Item: Over the past 2 weeks, how often have you been bothered by the following problems: Feeling nervous, anxious, or on edge? Response scale: 0 (not at all) to 3 (nearly every day) Scoring & interpretation: responses to the items are summed; possible scores range from 0–6; a score greater than 3 indicates likelihood of anxiety disorder
Health and Functioning	Self-rated health	SONNET survey	1	Psychometric information: Item: In general, would you say your health is…” Response scale: 1 (excellent) to 5 (poor) Scoring & interpretation: lower score indicates better self-rated health
Health and Functioning	Functioning	SONNET survey	2	Psychometric information: Example Item: In the past 30 days, for how many days were you totally unable to carry out your usual activities or work because of any health condition? Response scale: number of days 0–30 Scoring & interpretation: Each item is scored individually; higher number of days indicates greater functioning limitations
Health and Functioning	Coping	Brief Coping Orientation to Problems Experienced (COPE; selected subscales) [26, 27]	12	Psychometric information: Example Item: I’ve been getting emotional support from others Response scale: 1 (I haven’t been doing this at all) to 4 (I’ve been doing this a lot) Scoring & interpretation: Subscales include: Active coping, Emotional support, Behavioral disengagement, Use of informational support, Positive reframing, and Planning. Scores for each of the two items in subscale are totaled, with higher scores indicating greater orientation to that coping reaction
			54	TOTAL

*SONNET* Social Needs Network for Evaluation and Translation https://www.kpwashingtonresearch.org/our-research/research-areas/social-determinants/sonnet

The survey was administered in English and Spanish using a mixed mode (email, hard copy, telephone) approach. Data collection occurred at two timepoints: a “baseline” survey administered within one month of the patient’s qualifying primary care encounter and a follow up survey conducted approximately 3 months later. Eligible patients received a notification letter with $2 bill pre-incentive at each data collection period with information about the project, including the URL to the web-based survey. Patients who did not opt out of the evaluation, and for whom we had valid email addresses, received one initial email invitation followed by three reminder emails with an authenticated link to the survey. Finally, patients could complete a telephone interview of the survey. All patients received $20 and $25 cash for the baseline and follow up surveys, respectively.

#### Primary outcomes: patient social needs and self-efficacy

The primary outcomes in this study were patient ease/difficulty following their care plan, social risk (food, housing, transportation, utilities, debt, social isolation), perceived burden of social risk, and social needs. The YCLS measure provided patient information on social risk and needs; an additional team-generated item assessed perceived burden of social needs.

#### Patient coping, symptoms, and functioning

Participant self-rated health and functioning were assessed using items developed by the World Health Organization (WHO) [28]. Participant mental health and wellbeing were assessed using the Patient Health Questionnaire-2 (PHQ-2) [24] and the Generalized Anxiety Disorder-2 (GAD-2) [25]. Coping reactions were measured using validated subscales from the Brief Coping Orientation to Problems Experienced instrument [26, 27]. Self-efficacy was measured using the General-Self Efficacy Scale (GSE-6) [22].

#### Patient engagement and healthcare utilization

Two patient experience constructs were assessed–patient trust in the care team and patient-care team alliance. Trust in care team was assessed using a single item from a previous survey utilized in a KP SONNET study [20], and alliance was assessed using the Working Alliance Inventory Short Form (WAI-S) [23]. The team developed a measure to assess the value of the CRS.

We explored healthcare utilization in terms of five metrics, comparing 3 months before the qualifying encounter and 3 months after: (1) number of urgent care encounters; (2) number of inpatient hospital stays; (3) number of no shows for scheduled appointments; (4) number of same-day cancellations; and (5) total number of encounters.

### Quantitative analysis plan

To examine differences between responders and non-responders to the baseline survey, we performed Cramer’s V equivalence tests across the three groups (CRS-S, CRS-M, PSC) on basic demographics with a very conservative upper caliper limit of V = 0.15. Missing data were explored and described. To compare the groups on change over time, we computed longitudinal Generalized Estimating Equations (GEE) using partially nested models (CRS group nested within individual CRS, baseline and follow-up timepoints were nested within patient). Full maximum likelihood estimation with robust covariance matrix parameter estimates was used to obtain coefficients and statistically account for missing data. Count-based dependent variables were analyzed using a Poisson distribution with a log link, and dichotomous dependent variables were estimated using a binomial distribution with a logit link. All analyses controlled for baseline differences among the groups using the three-stage propensity score selection and adjustment process (described in the Supplementary file 1), as well as the longitudinal GEE. Therefore, all statistics presented are estimates adjusting for baseline characteristics and scores on each variable.

### Power analysis

We conducted an a priori power analysis for the effectiveness evaluation. We estimated effect size variability at the clinic level to range from 0.05 to 0.25, and aimed to detect a Cohen’s d effect size of 0.25 to 0.40 on primary outcomes of interest. We assumed an attrition rate of approximately 25% from baseline to follow-up. Power analyses indicated a need to recruit approximately 30 patients at each of the 32 clinics (recruit 1200 patients to retain 900), with 10 patients at each clinic in each group: CRS-S, CRS-M, PSC.

### Qualitative evaluation participants

The qualitative evaluation collected data on patient experience. Patients were recruited from three distinct groups— CRS-M patients, the CRS-S group and the PSC group. Since a goal of the qualitative evaluation, conducted at the behest of KPWA, was to characterize a successful CRS-patient relationship, we asked the CRSs from 12 clinics to refer 3–5 patients who they felt exemplified a “success". Conversely, CRS-S and PSC patients were identified with the same broad parameters used in the survey: i.e., they reported either having social needs or difficulties fulfilling their care plan and either received one or no CRS encounters, respectively. The CRS-S and PSC patients were interviewed to include patients that may have had barriers to accessing CRS support or developing a successful relationship as well as patients that might have had different levels of need for support. All patients were sent a letter introducing the project and offered a number to call if they wished to participate. If no call was received within 7–10 days, project staff conducted recruitment calls; patients could opt out at any time. All patient interviews were conducted via phone. All interviews were recorded and transcribed. Patients were mailed $50 cash to thank them for their participation.

### Qualitative analysis plan

We selected the Rapid Group Analysis Process (Rap-GAP) [29]. method for its speed and ability to include a variety of health system and research partners which enhanced understanding and buy in of the results. In this pragmatic QI evaluation, health system leaders were awaiting results to inform decision-making, so there was limited time for qualitative analysis of the 45 interviews. Rap-GAP uses a team of study staff and/or collaborators to review and annotate transcripts for key ideas and insights. Participants in our process included nine members of the study team. Participants were assigned 4–5 transcripts and were asked to note key insights the interview data provided and associate these with one or more quotes. Domains of interest included: 1) Benefits of CRS to patients, 2) Satisfaction with CRS, 3) Improvements for the CRS role, 4) best practice examples, 5) Reflections on KP, 6) Other insights or themes that are surprising or important. Individually annotated ideas and insights were transferred to a virtual white board app as sticky notes and placed in their corresponding domain of interest. The group then worked together during a facilitated 2-h discussion session to organize the sticky notes into tagged themes. The clustered sticky notes were then exported to a spreadsheet where they were sorted by tag/theme and the quotes associated with individual ideas were copied. This spreadsheet was then used for the final synthesis. Key findings were summarized into coding memos and tables, and then discussed in an iterative process with the Rap-GAP team.

### Mixed methods analysis

In accordance with the use of a sequential mixed methods approach [21], we conducted our quantitative data collection and analysis followed by qualitative data collection and analysis with the goal of using qualitative data to expand on quantitative findings. Also, the quantitative data was used to identify people for the CRS-S and PSC patient interviews. Once both data sources were analyzed the team went through a process looking at both sets of data with the aim of identifying areas where the qualitative helped enhance or expand understanding of the quantitative data or where the qualitative raised new issues or insights not encompassed by the quantitative data. This exercise resulted in a summary report that was presented to the delivery system as well as the findings in this paper.

## Results

### Patient demographics

**Fig. 1:**
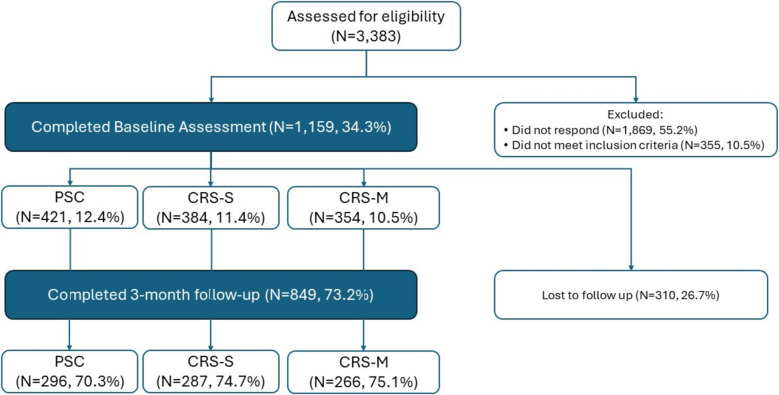
Consort Diagram. This figure depicts the number of patients for whom we received surveys at each timepoint across the three arms: (1) Propensity Score Comparison (PSC) arm; (2) Community Resource Specialist– Single Session (CRS-S); (3) CRS– Multiple Session (CRS-M), as well as those excluded and lost to follow up

 See Fig. 1 for Consort Diagram inclusive of response rates for both survey timepoints. Response rates at 3-month follow up were statistically different among the groups; see Table 2 for full details of demographics by group. Cramer’s V equivalence tests found no differences among baseline responder and non-responder groups. Patients were primarily female (72.8%), White (78.9%), and had Medicare (57.5%), with a mean age of 62.3 (*SD* = 17.6). At baseline there were differences between groups with respect to patient sex, race, and insurance type.

**Table 2:** Patient demographics

	**PSC** **M(SD)/n(%)**	**CRS-S** **M(SD)/n(%)**	**CRS-M** **M(SD)/n(%)**	**Total** **M(SD)/n(%)**	**Non-respondents** **M(SD)/n(%)**
Age	62.1 (16.3)	61.7 (18.5)	63.0 (17.9)	62.3 (17.6)	63.4 (18.3)
Sex^a^					
Female	321 (76.2%)	281 (73.2%)	242 (68.4%)	844 (72.8%)	1555 (69.4%)
Race^a^					
Asian	7 (1.7%)	14 (3.6%)	10 (2.8%)	31 (2.7%)	96 (4.3%)
Black or African American	44 (10.5%)	23 (6.0%)	19 (5.4%)	86 (7.4%)	231 (10.3%)
Native Hawaiian/Pacific Islander	3 (.7%)	2 (.5%)	1 (.3%)	6 (.5%)	20 (9%)
American Indian/Alaskan Native	15 (3.6%)	10 (2.6%)	9 (2.5%)	34 (2.9%)	65 (2.9%)
Other	25 (5.9%)	16 (4.2%)	8 (2.3%)	49 (4.2%)	79 (3.5%)
Unknown or not reported	9 (2.1%)	12(3.1%)	17 (4.8%)	38 (3.3%)	126 (5.6%)
White	318 (75.5%)	307(79.9%)	290 (81.9%)	915 (78.9%)	1625 (72.5%)
Latinx					
No	385 (91.4%)	339 (88.3%)	319 (90.1%)	1043 (90.0%)	1993 (88.9%)
Unknown	8 (1.9%)	17 (4.4%)	17 (4.8)	42 (3.6%)	122 (5.4%)
Yes	28 (6.7%)	28 (7.3%)	18 (5.1%)	74 (6.4%)	127 (5.7%)
Insurance type^a^					
Commercial	62 (14.7%)	86 (22.4%)	58 (16.4%)	206 (17.8%)	387 (17.3%)
High-deductible	8 (1.9%)	16 (4.2%)	9 (2.5%)	33 (2.8%)	60 (2.7%)
Medicare	276 (65.6%)	202 (52.6%)	189 (53.4%)	667 (57.5%)	1289 (57.5%)
Molina Medicaid	13 (3.1%)	11 (2.9%)	20 (5.6%)	44 (3.8%)	65 (2.9%)
Not enrolled	35 (8.3%)	52 (13.5%)	54 (15.3%)	141 (12.2%)	316 (14.1%)
Subsidized ACA	24 (5.7%)	14 (3.6%)	17 (4.8%)	55 (4.7%)	91 (4.1%)
Unsubsidized ACA	3(.7%)	3 (.8%)	7 (2.0%)	13 (1.1%)	34 (1.5%)

^a^Chi-square test <.05. Cramer’s V of 0.15 or lower reflect equivalence. All Cramer’s V for the table were less than 0.09

### Primary outcomes

At the 3-month follow-up (controlling for baseline scores and differences among the groups), there were few differences among the groups in terms of our primary outcomes; see Table 3. The CRS-M group was more likely than the CRS-S group to report food insecurity (34% vs. 24%, *p* = 0.019) and less likely to report utilities insecurity (11% vs. 17%, *p* = 0.049). The CRS-M group was less likely than the PSC group to report debt insecurity (25% vs. 31%, *p* = 0.008). The CRS-M group rated their social risk severity higher than the PSC group (M = 6.9 vs. 6.3, *p* = 0.016). There were no differences among the groups for: social isolation, housing insecurity, transportation issues, total number of social risk domains, number of social needs, or difficulty following through with their care plan.

**Table 3:** Primary and secondary outcomes comparing all groups using estimated values from generalized estimating equations

	CRS-M		CRS-S		PSC		Significant model comparison
	M	SE	M	SE	M	SE	
Primary Outcomes							
Food insecurity	0.34	0.03	0.24	0.03	0.28	0.03	(A) 0.019
Housing insecurity	0.31	0.04	0.34	0.03	0.33	0.04	NS
Transportation insecurity	0.20	0.03	0.20	0.02	0.24	0.03	NS
Utilities insecurity	0.11	0.02	0.17	0.02	0.11	0.02	(A) 0.049
Debt insecurity	0.20	0.03	0.25	0.03	0.31	0.03	(B) 0.008
Social Isolation	2.47	0.08	2.29	0.07	2.45	0.07	NS
# of social risks	2.06	0.14	1.83	0.13	1.99	0.12	NS
# of social needs	2.60	0.17	2.46	0.17	2.31	0.15	NS
Severity of social risk	6.94	0.17	6.74	0.21	6.34	0.17	(B) 0.016
Ease/difficulty with care plan	1.79	0.05	1.91	0.05	1.83	0.05	NS
Patient Experience with Care Team							
Trust	8.83	0.09	8.55	0.11	8.63	0.10	(A) 0.033
Working Alliance: Task	21.98	0.28	20.74	0.32	21.58	0.28	(A) 0.004
Working Alliance: Bond	23.26	0.29	22.14	0.35	22.65	0.29	(A) 0.012
Working Alliance: Goal	21.67	0.29	20.83	0.33	22.06	0.29	(C) 0.007
Health and Functioning							
Self-Rated Health	3.41	0.06	3.35	0.06	3.22	0.06	(B) 0.028
General Self-Efficacy	2.99	0.03	3.01	0.03	3.01	0.03	NS
Help with Tasks for Daily Living	1.92	0.06	1.75	0.05	1.68	0.05	(A) 0.042 (B) 0.002
Days totally unable to carry out activities	4.84	0.49	5.08	0.47	3.88	0.40	NS
Days partially unable to carry out activities	7.01	0.57	6.76	0.52	6.67	0.48	NS
Depression	1.71	0.11	1.67	0.11	1.65	0.11	NS
Anxiety	1.75	0.11	1.65	0.11	1.89	0.11	NS
Active Coping	3.19	0.05	3.20	0.05	3.32	0.04	NS
Use of Emotional Support	2.78	0.06	2.82	0.06	2.99	0.05	(B) 0.008 (C) 0.031
Behavioral Disengagement	1.78	0.05	1.66	0.05	1.64	0.05	(B) 0.048
Instrumental Support	2.69	0.06	2.70	0.05	2.91	0.05	(B) 0.004 (C) 0.005
Positive Reframing	2.95	0.05	3.03	0.05	3.12	0.05	(B) 0.022
Planning	3.46	0.21	3.30	0.20	3.55	0.20	NS
Healthcare Utilization—Proportion							
Urgent care encounters	0.24	0.02	0.22	0.02	0.18	0.02	(B) 0.048
Inpatient stay	0.05	0.01	0.06	0.01	0.03	0.01	NS
No shows	0.28	0.03	0.22	0.03	0.13	0.02	(B) <.001
Same-day cancellations	0.21	0.03	0.25	0.03	0.16	0.02	(C) 0.010

Values derived from Generalized Estimating Equations controlling for baseline characteristics and variable score

A = CRS-M vs. CRS-S; B = CRS-M vs. PSC; C = CRS-S vs. PSC, *NS* Not significant at *p* <.05. Values derived from Generalized Estimating Equations controlling for baseline characteristics and variable score

### Health and functioning

At the three month follow-up, the CRS-M group had significantly lower scores than the control group on several coping skills subscales, including Emotional Support (M = 2.8 vs. 3.0, *p* = 0.008), Use of Instrumental Support (M = 2.7 vs. 2.9, *p* = 0.004) and Positive Reframing (M = 3.0 vs. 3.1, *p* = 0.022), and higher scores on Behavioral Disengagement (M = 1.8 vs. 1.6, *p* = 0.048). The CRS-S group had significantly lower scores than the PSC group on Emotional Support (M = 2.8 vs. 3.0, *p* = 0.031) and Use of Instrumental Support (M = 2.7 vs. 2.9, *p* = 0.005). There were no differences among the groups in the Active Coping or Planning subscales.

The CRS-M group’s self-rated health scores were significantly worse than the PSC group (M = 3.4 vs. 3.2, *p* = 0.028) and they endorsed needing more help with tasks for daily living than both the CRS-S group (*p* = 0.042) and the PSC group (M = 1.92 vs. 1.75 and 1.68, *p* = 0.002). There were no differences among the groups in their PHQ-2 or GAD-2 scores, or the impact of their health on activities or work, or on self-efficacy.

### Patient experience with the care team

The CRS-M group reported higher satisfaction with the CRS than the CRS-S group (M = 3.9 vs. 3.8, *p* = 0.023), and higher overall levels of trust in their KP healthcare providers (M = 8.8 vs. 8.6, *p* = 0.033). The CRS-M group reported higher scores than the CRS-S group on the Working Alliance Task Subscale (M = 22.0 vs. 20.7, *p* = 0.004) and Bond Subscale (M = 23.3 vs. 22.1, *p* = 0.012). The CRS-S group had lower scores than the PSC group on the Working Alliance Goal subscale (M = 20.8 vs. 22.1, *p* = 0.007).

### Healthcare utilization

A higher proportion of patients in the CRS-M group than in the PSC group had an urgent care encounter (24% vs. 18%, *p* = 0.048), canceled an appointment over a day before it was scheduled to occur (53% vs. 40%, *p* = 0.039), and did not show up for their appointments (28% vs. 13%, *p* < 0.001). A higher percentage of CRS-S group than the PSC group canceled on the same day as their appointment (25% vs. 16%, *p* = 0.010).

### Qualitative findings

Recruitment efforts resulted in interviews with 30 CRS-M patients with “successful relationships” with the CRS, 7 CRS-S patients and 8 PSC patients. Qualitative findings from patients centered on their experience working with the CRS, including sharing out about resources provided by the CRS, and about subsequent improvements to their overall health and well-being. Patients shared that CRSs provided them with referrals to a wide variety of resources, the most often mentioned included; 1) financial assistance (e.g., KP’s Medical Financial Assistance program), 2) food assistance, 3) mental health care, 4) government programs, 5) senior services (e.g., assistance finding a caregiver), 6) access to transportation, and 7) help with utilities. Patients credited the CRS with improving their overall health, making statements like “she saved my life,” “I feel like I got my life back” and “[she] gave me a sense of hope.” Patients cited benefits such as decreased financial stress, increased ability to fulfill their care plan and access care, as well as satisfaction with the CRS and how the role positively reflected on KP. Illustrative patient quotes are captured in Table 4.

**Table 4:** Exemplar quotes reflecting themes from patient qualitative interviews

Theme	Exemplar CRS Patient Quotes^*^
Improvements in emotional well-being and mental health	*It's a struggle when you have so many chronic things happening and having to see a doctor just to find out why you have so much problems. How can you manage it on your own? It makes it hard, it makes it more difficult, so having somebody that says we have this program that has these services…you fill that out, you feel a relief from all that pressure and stressing over not being able to do that because you're limited. You already have so many chronic problems, and then stressing over something– it's wonderful to have services like that*
	*Well, psychologically it was a big help just being able to have the paratransit take me places. It was something I looked forward to, it give me a chance to actually get out and about a little bit*
Decreased financial stress	*Oh, a lot. I mean, she saved my life. I couldn't afford it. I couldn't have done it. And I had a high option, and after all those years and years of paying it came down to that*
	*I was at the end of my rope before I found out about all this stuff. I was crying… I have to pay $1,000 almost a month for my [medication], it's that bad. I only make about $1,600 a month*
	*I had no idea that Kaiser had this program with the MFA until I happened to call one day and spoke with somebody, her name is Tammy, she works up front as receptionist and she's the one that got me connected with Dena. I don't know the exact words to express the world of difference for me, because I have a lot of prescriptions. I wouldn't say I'm sickly, but I have bad allergies, I have asthma, I have things I have to have in my routine day-to-day and they would be very costly for me and frankly, I don't know if I would be able to afford them without the MFA—that has just been a night and day difference just to know I have that available to me*
Satisfaction with the CRS role and perceptions of KP	*So yeah, she really did above and beyond just getting me connected with resources and the MFA status, it was like talking to one of my friends on the phone or meeting with one of my friends when I had stuff going on. I recommended her to several of my coworkers*
	*CRS is a wonderful program. I would definitely 100% recommend it, support it, advocate for it, because I think that a lot of people are in need of it, they just don't know the program exists, how there is this service that can help*
	*I don't know what I'd do, where I'd be without her.“ I'm very pleased, beyond satisfied. I'm encouraged because there is direction, there are people who can be part of the pursuit that I want, I just have to find them, and she has definitely opened doors. Not everybody does that. You can still be professional and do your job in your role according to your values, which may be different from mine. Finding somebody that will help me take the next steps in my terms is challenging. But [the CRS]has definitely been very helpful*
	*Well, since I've been with Kaiser for over 30 years and never met anyone I could approach with a situation and get it resolved…. This is the first time*
Coordination of care and advocacy	*I still have problems with the referral with other doctors, really because of fear. I'm working on it, but I'm not going to promise anything on that one. If [the CRS] has her way, she'll cart me off to the cardiologist, she's like, you're going to make that appointment, girl*
	*I have recurring UTI's, very frequent, so therefore I'm on antibiotics all the time…I’ve had them long enough to know what's happening. I know what it is, and I know what I need. I need a urinalysis and the antibiotic, quick. I called about 7:30 in the morning. They said the best I can do would be this afternoon, around 4:30 or 5 for the phone visit. I said no, that's not soon enough…I had to go…so I hung up. Well, about 10 min later [the CRS] called. She said,"I see something is going on here, can I help you?"I said no, I just have to wait, I'm trying to get a phone visit. She says I'll call you back. And she called back in a little bit and said I had one at 9 something that morning. See, that's what I mean. I can go to her and she helps in a time of my crisis*
Barrier to use: pride and privacy	*I would rather those types of services [CRS SERVICES] be available to people that really need them. That's why I wouldn't have any interest in potentially taking services away from people who really need them*
	*We're very private about that type of stuff. We don't tell people we have issues. So there's really no reason anybody would offer assistance along those lines, because we just don't make that type of stuff known*

^*^Most of these themes were only relevant to people who had at least one CRS encounter, but PSC patient quotes were included where appropriate

PSC patients reported having social needs that they felt able to manage on their own, or with other resources within the healthcare system such as by working with a mental health provider. They also referenced utilizing community support and organizations such as churches and senior living resources. Even so, there were a number of missed opportunities for CRS referral uncovered during interviews. Some patients reported that they wanted help setting health goals and that they could use the support from a CRS once the role was described to them. Yet, many PSC patients shared that they did not want help from the health system with these particular issues, they felt a strong sense of pride and a high need for privacy, and that others needed the services more than they did.

## Discussion

This pragmatic quality improvement evaluation of Community Resource Specialist (CRS) integration in 32 KPWA primary care clinics to address social needs revealed mixed results. The quantitative data suggest that patients who worked with the CRS for multiple sessions were worse off in social risk severity and food insecurity, but improved for financial risks such as utilities and debt insecurity. For the majority of primary and secondary outcomes (e.g., social isolation, housing, difficulties following a care plan, depression, anxiety), there were no differences across groups after three months. Interestingly, patients with multiple CRS sessions demonstrated poorer coping in several areas, whereas PSC patients showed higher use of instrumental and emotional support coping strategies which might explain why they did not meet with the CRS. Overall, patients who had several CRS sessions reported having worse health and needing more help with activities of daily living, suggesting these patients might be more complex, which was confirmed with post hoc analyses exploring several comorbidity indicators, and their utilization patterns (e.g., more urgent care encounters, canceled appointments, and no-shows). The qualitative data confirmed that the CRS-M patients with successful CRS relationships often had quite complex health needs, but added the insight that patients reported that the CRS had a very positive impact across almost all outcomes of interest, including social risk and mental health This focus on positive impact is likely due to our sampling approach to oversample successful patient-CRS relationships. Even so, no patients reported improvements in their physical health in the qualitative interviews, which is consistent with the findings from the quantitative analyses, and the broader literature, including the original PCORI-funded pilot study that led to the genesis of the CRS role [12].

Subsequent to completion of our study, a Comprehensive Conceptual Model for Social Care Interventions offered four pathways for patient improvement one of which may explain our findings [17]. Indeed, there is evidence to suggest that roles like the CRS improve the patient experience with the healthcare system, which might mediate healthcare outcomes if in place and measured on a longer time scale [30–34]. In this study, we assessed trust in the care team; patients who had multiple encounters with the CRS showed improvements in trust over time. Moreover, we used the Working Alliance Inventory [23], which consists of three scales, two of which (i.e., *bond*, *task*) were enhanced over time in patients with multiple CRS encounters. CRSs are encouraged to build connections in the local community and the primary care team, functioning as a bridge to both that can engender trust in patients [35]. Our quantitative findings lend support for the “emotional support/healing relationships pathway” of this updated social care logic model [17]. Our qualitative data offer convergent validity for this pathway, but also for the health care services connections pathway of the logic model [17]. Indeed, key themes from patient interviews reflected improvements in emotional well-being, decreased stress, and care coordination and advocacy. Simultaneously, this logic model might provide an explanation for self-reported increased needs for patients with multiple CRS encounters: patients who grew to trust their CRS might have felt more comfortable disclosing new social needs [36]. Yet, studies comparing patient navigation to a social health focused CRS (or community health worker) are needed to explore whether the roles are unique, redundant, or complementary. Studies that explicitly test these pathways longitudinally are critically needed.

KPWA did not universally screen for social needs during this evaluation because they did not have the staffing or technological resources. This led to several compromises to the evaluation of the quality improvement initiative, but also perhaps undermined the start of the CRS-patient relationship. Interviews with PSC patients suggested that some might have benefited from the CRS and went undetected because their care team did not systematically screen for social risks. Yet, not all PSC patients wanted help with social risks from the healthcare system, reaffirming the importance of distinguishing between social risk and needs [37, 38]. Since this evaluation ended, KPWA piloted universal social health screening upon Epic’s release of their Social Determinants of Health module in two clinics. With mounting literature suggesting the profound prevalence and impact of social risk and needs on a person’s physical health, even among more privileged populations, quality metrics [39, 40] are emerging to encourage proliferation of both screening and intervention with guidance from the National Academy of Science, Engineering, and Medicine [41].

### Limitations

Importantly, there are several design issues associated with this pragmatic quality improvement evaluation, many of which point to important areas of inquiry for future research. First, this evaluation had no true baseline assessment of any variables given that patients entered into the evaluation after having a qualifying encounter. Although the survey team tried to reach the patient as soon after the qualifying encounter as possible, they were granted up to one month to make contact for the initial survey. Patients might have already benefited from the intervention or experienced worsening of symptoms in the interim. Second, the evaluation team was working concurrently with the PF team and the two bodies of work could not be synced due to delayed changes to the EHR. Therefore, the CRS integration in the primary care team was not optimized when the evaluation began. Third, the length of follow up may be insufficient to observe meaningful and sustainable changes in most constructs. Fourth, our attempt to use propensity scores to generate a matched comparator was insufficient and a more robust design that includes randomization would be ideal. Fifth, the qualitative sampling strategy favored delivery system wishes to feature success stories, which biases the results reported herein. Finally, the measures of healthcare utilization were not comprehensive and there is emerging evidence to suggest that these types of interventions can lead to appropriate increases in primary care, for instance [42].

## Conclusion

This quality improvement evaluation of a new primary care team role—the Community Resource Specialist (CRS)—represents one way health systems can respond to growing calls to screen for and address patient social needs. Although the quantitative evaluation revealed limited positive impact on financial risks, apparent worsening of food insecurity, and no impact on mental or physical health after three months, the impact of having multiple CRS encounters was clear in terms of improving trust in the care team. The qualitative findings underscore the CRS potential to activate both an emotional support/healing relationship pathway and a health care services pathway. Emergent from this study is a playbook with practical steps, exercises, and other resources to equip other healthcare settings to do this work [43]. More rigorous designs and longer-term follow up are needed to explore whether these pathways activated by social health interventions indeed lead to improvements in patient physical health and health care system utilization.

## Supplementary Information

Supplementary Material 1.

Supplementary Material 2.

Supplementary Material 3.

## Data Availability

The datasets generated and/or analysed during the current study are not publicly available due to this being a quality improvement study, but may be available from the corresponding author on reasonable request.

## References

[1] Braveman P, Egerter S, Williams DR. The social determinants of health: coming of age. Annu Rev Public Health. 2011;32(1):381–98. 10.1146/annurev-publhealth-031210-101218. 21091195 10.1146/annurev-publhealth-031210-101218

[2] Kaplan GA, Shema SJ, Leite CM. Socioeconomic determinants of psychological well-being: the role of income, income change, and income sources during the course of 29 years. Ann Epidemiol. 2008;18(7):531–7. 10.1016/j.annepidem.2008.03.006. 18504142 10.1016/j.annepidem.2008.03.006 PMC2771109

[3] Marmot M. Social determinants of health inequalities. Lancet. 2005;365(9464):1099–104 10.1016/s0140-6736(05)71146-6. 15781105 10.1016/S0140-6736(05)71146-6

[4] De Marchis EH, Alderwick H, Gottlieb LM. Do patients want help addressing social risks? J Am Board Fam Med. 2020;33(2):170–5. 10.3122/jabfm.2020.02.190309. 32179597 10.3122/jabfm.2020.02.190309

[5] Cottrell EK, Hendricks M, Dambrun K, Cowburn S, Pantell M, Gold R, Gottlieb LM. Comparison of community-level and patient-level social risk data in a network of community health centers. JAMA Netw Open. 2020;3(10):e2016852. 10.1001/jamanetworkopen.2020.16852. 33119102 10.1001/jamanetworkopen.2020.16852 PMC7596576

[6] Gottlieb LM, Wing H, Adler NE. A systematic review of interventions on patients’ social and economic needs. Am J Prev Med. 2017;53(5):719–29. 10.1016/j.amepre.2017.05.011. 28688725 10.1016/j.amepre.2017.05.011

[7] Bryant A, Walsh-Felz A, Jacklitz J, Lindberg S. The impact of a community resource navigator program on patient trust. WMJ. 2020;119(3):190–3. 33091287 PMC9125772

[8] Kangovi S, Mitra N, Grande D, et al. Patient-centered community health worker intervention to improve posthospital outcomes: a randomized clinical trial. JAMA Intern Med. 2014;174(4):535–43. 10.1001/jamainternmed.2013.14327. 24515422 10.1001/jamainternmed.2013.14327

[9] Davidson KW, McGinn T. Screening for social determinants of health: the known and unknown. JAMA. 2019;322(11):1037–8. 10.1001/jama.2019.10915. 31465095 10.1001/jama.2019.10915

[10] Weintraub D, Rodgers MA, Botcheva L, et al. Pilot study of medical-legal partnership to address social and legal needs of patients. J Health Care Poor Underserved. 2010;21(2 Suppl):157–68. 10.1353/hpu.0.0311. 20453383 10.1353/hpu.0.0311

[11] National Committee for Quality Assurance. HEDIS MY 2023 Measure description. National Committee for Quality Assurance. https://www.ncqa.org/wp-content/uploads/2022/07/HEDIS-MY-2023-Measure-Description.pdf. Accessed 19 Aug 2024.

[12] Hsu C, Hertel E, Johnson E, et al. Evaluation of the learning to integrate neighborhoods and clinical care project: findings from implementing a new lay role into primary care teams to address social determinants of health. Perm J. 2018;22. 10.7812/tpp/18-101. 10.7812/TPP/18-101 PMC6207445 32392126

[13] Adding a New Role at Clinics to Help Patients Access Community Resources. Patient-centered outcomes research institute. 2024. https://www.pcori.org/research-results/2012/adding-new-role-clinics-help-patients-access-community-resources. Accessed 19 Aug 2024. 37782706

[14] Olaniran A, Smith H, Unkels R, Bar-Zeev S, van den Broek N. Who is a community health worker? A systematic review of definitions. Glob Health Action. 2017;10(1):1272223. 10.1080/16549716.2017.1272223. 28222653 10.1080/16549716.2017.1272223 PMC5328349

[15] Larson EB. Learning Health System initiative weaves research into care delivery at KP Washington. Healthy Findings blog. 2017. https://www.kpwashingtonresearch.org/news-and-events/blog/2017/jul-2017/Learning_Health_System_initiative_weaves_research_into_care_delivery_at_KP_Washington.

[16] Allen C, Coleman K, Mettert K, Lewis C, Westbrook E, Lozano P. A roadmap to operationalize and evaluate impact in a learning health system. Learn Health Syst. 2021;5(4): e10258. 10.1002/lrh2.10258. 34667878 10.1002/lrh2.10258 PMC8512726

[17] Gottlieb LM, Hessler D, Wing H, Gonzalez-Rocha A, Cartier Y, Fichtenberg C. Revising the Logic Model Behind Health Care’s Social Care Investments. Milbank Q. 2024;102(2):325–35. 10.1111/1468-0009.12690. 38273221 10.1111/1468-0009.12690 PMC11176407

[18] LaForge K, Gold R, Cottrell E, et al. How 6 organizations developed tools and processes for social determinants of health screening in primary care: an overview. J Ambul Care Manage. 2018;41(1):2–14. 10.1097/JAC.0000000000000221. 28990990 10.1097/JAC.0000000000000221 PMC5705433

[19] Sundar KR. Universal Screening for Social Needs in a Primary Care Clinic: A Quality Improvement Approach Using the Your Current Life Situation Survey. Perm J. 2018;22:18–089. 10.7812/TPP/18-089. 30296397 10.7812/TPP/18-089 PMC6175598

[20] Lewis CC, Wellman R, Jones SMW, et al. Comparing the performance of two social risk screening tools in a vulnerable subpopulation. J Family Med Prim Care. 2020;9(9):5026–34. 10.4103/jfmpc.jfmpc_650_20. 33209839 10.4103/jfmpc.jfmpc_650_20 PMC7652127

[21] Palinkas LA, Aarons GA, Horwitz S, Chamberlain P, Hurlburt M, Landsverk J. Mixed method designs in implementation research. Adm Policy Ment Health. 2011;38(1):44–53. 10.1007/s10488-010-0314-z. 20967495 10.1007/s10488-010-0314-z PMC3025112

[22] Romppel M, Herrmann-Lingen C, Wachter R, Edelmann F, Dungen HD, Pieske B, Grande G. A short form of the General Self-Efficacy Scale (GSE-6): development, psychometric properties and validity in an intercultural non-clinical sample and a sample of patients at risk for heart failure. Psychosoc Med. 2013;10:Doc01. 10.3205/psm000091. 10.3205/psm000091 PMC3578200 23429426

[23] Hatcher RL, Gillaspy JA. Development and validation of a revised short version of the Working Alliance Inventory. Psychother Res. 2006;16(1):12–25 10.1080/10503300500352500.

[24] Kroenke K, Spitzer RL, Williams JB. The patient health questionnaire-2: validity of a two-item depression screener. Med Care. 2003;41(11):1284–92. 10.1097/01.MLR.0000093487.78664.3C. 14583691 10.1097/01.MLR.0000093487.78664.3C

[25] Kroenke K, Spitzer RL, Williams JB, Monahan PO, Lowe B. Anxiety disorders in primary care: prevalence, impairment, comorbidity, and detection. Ann Intern Med. 2007;146(5):317–25. 10.7326/0003-4819-146-5-200703060-00004. 17339617 10.7326/0003-4819-146-5-200703060-00004

[26] Carver CS. You want to measure coping but your protocol’s too long: consider the brief COPE. Int J Behav Med. 1997;4(1):92–100. 10.1207/s15327558ijbm0401_6. 16250744 10.1207/s15327558ijbm0401_6

[27] Carver CS, Scheier MF, Weintraub JK. Assessing coping strategies: a theoretically based approach. J Pers Soc Psychol. 1989;56(2):267–83. 10.1037/0022-3514.56.2.267. 2926629 10.1037//0022-3514.56.2.267

[28] Ustun TB, Kostanjesek N, Chatterji S, Rehm J, World Health Organization, eds. Measuring health and disability: manual for WHO Disability Assessment Schedule (WHODAS 2.0). World Health Organization; 2010. https://www.who.int/publications/i/item/measuring-health-and-disability-manual-for-who-disability-assessment-schedule-(-whodas-2.0).

[29] Hsu C, Mogk J, Hansell L, Glass JE, Allen C. Rapid group analysis process (Rap-GAP): a novel approach to expedite qualitative health research data analysis. Int J Qual Methods. 2024;23:16094069241256276. 10.1177/16094069241256275. 10.1177/16094069241256275 PMC12753008 41477558

[30] Bell H, Brower H, Chepaitis A, et al. Accountable Health Communities (AHC) model evaluation, second evaluation report. 2023. p. 386. https://www.cms.gov/priorities/innovation/data-and-reports/2023/ahc-second-eval-rpt.

[31] Review of Evidence for Health-Related Social Needs Interventions. The Commonwealth Fund. https://www.commonwealthfund.org/sites/default/files/2019-07/COMBINED-ROI-EVIDENCE-REVIEW-7-1-19.pdf. Accessed 19 Aug 2024.

[32] Johnson SL, Gunn VL. Community health workers as a component of the health care team. Pediatr Clin North Am. 2015;62(5):1313–28. 10.1016/j.pcl.2015.06.004. 26318954 10.1016/j.pcl.2015.06.004

[33] Islam N, Nadkarni SK, Zahn D, Skillman M, Kwon SC, Trinh-Shevrin C. Integrating community health workers within patient protection and affordable care act implementation. J Public Health Manag Pract. 2015;21(1):42–50. 25414955 10.1097/PHH.0000000000000084 PMC4416641

[34] Payne J, Razi S, Emery K, Quattrone W, Tardif-Douglin M. Integrating community health workers (CHWs) into health care organizations. J Community Health. 2017;42(5):983–90. 10.1007/s10900-017-0345-4. 28391593 10.1007/s10900-017-0345-4

[35] Herman AA. Community health workers and integrated primary health care teams in the 21st century. J Ambul Care Manage. 2011;34(4):354–61. 21914991 10.1097/JAC.0b013e31822cbcd0

[36] Mahmud A, Brown MC, Wong ES, et al. Comparison of clinic-based assistance versus a centralized call center on patient-reported social needs: findings from a randomized pilot social health integration program. BMC Public Health. 2025;25(1):1171. 10.1186/s12889-025-22334-x. 40148873 10.1186/s12889-025-22334-x PMC11951525

[37] Alderwick H, Gottlieb LM. Meanings and misunderstandings: a social determinants of health lexicon for health care systems. Milbank Q. 2019;97(2):407–19. 10.1111/1468-0009.12390. 31069864 10.1111/1468-0009.12390 PMC6554506

[38] Tuzzio L, Wellman RD, De Marchis EH, et al. Social risk factors and desire for assistance among patients receiving subsidized health care insurance in a US-based integrated delivery system. Ann Fam Med. 2022;20(2):137–44. 10.1370/afm.2774. 35346929 10.1370/afm.2774 PMC8959745

[39] Hinton E, Diana A. Section 1115 Medicaid Waiver Watch: A Closer Look at Recent Approvals to Address Health-Related Social Needs (HRSN). KFF. Updated March 4, 2024. Accessed August 19, 2024. https://www.kff.org/medicaid/issue-brief/section-1115-medicaid-waiver-watch-a-closer-look-at-recent-approvals-to-address-health-related-social-needs-hrsn/

[40] Meyers DJ, Gadbois EA, Brazier J, Tucher E, Thomas KS. Medicare plans’ adoption of special supplemental benefits for the chronically ill for enrollees with social needs. JAMA Netw Open. 2020. 10.1001/jamanetworkopen.2020.4690. 32396191 10.1001/jamanetworkopen.2020.4690 PMC7218486

[41] National Academies of Sciences, Engineering, and Medicine. Integrating Social Care into the Delivery of Health Care: Moving Upstream to Improve the Nation's Health Integrating Social Care into the Delivery of Health Care Moving Upstream to Improve the Nation's Health. 2019. https://www.nap.edu/catalog/25467/integrating-social-care-into-the-delivery-of-health-care-moving 31940159

[42] Mahmud A, Wong ES, Lewis CC, et al. Differences in healthcare utilization across 2 social health support modalities: results from a randomized pilot evaluation. AJPM Focus. 2025;4(3): 100323. 10.1016/j.focus.2025.100323. 40242655 10.1016/j.focus.2025.100323 PMC12002760

[43] Community Resource Specialist Integration Playbook. https://www.act-center.org/application/files/8716/4859/5567/CRS_Playbook_Public.pdf.

