# Supplementary material for: Optimizing the community resource specialist to address social needs in primary care: results from a pragmatic quality improvement evaluation
Source: BMC Prim Care. 2025 Oct 31;26:330. doi: 10.1186/s12875-025-02922-x (PMC12577139; doi:10.1186/s12875-025-02922-x)
Supplement: Supplementary file 1 — Supplementary Material 1. [file 12875_2025_2922_MOESM1_ESM.docx]

**Supplementary File 1**

**Propensity Score Comparison Approach**

We used a three-stage, combined prospective and retrospective approach to propensity score generation. In the first stage, historical administrative data were analyzed using a propensity score modeling approach to identify demographic, diagnosis, and service use data associated with CRS service use. Because of our large sample size (*n* = 168,007) and the exploratory nature of the approach, we applied stepwise logistic regression with data from all KP sites using a “kitchen sink” approach to variable inclusion, predicting whether or not the patient had any CRS contact. We included 63 predictor variables such as demographics, clinic, types and frequency of treatment and service use, diagnoses, and insurance status. After removal of variables that did not significantly contribute to the model’s predictive ability, either due to lack of association or multicollinearity with stronger variables, the final model included 15 variables: clinic, insurance type, whether a behavioral health screen was conducted, number of outpatient visits, gender, race, cancer diagnosis, number of days as a KP member, depression diagnosis, received psychotherapy, frailty flag, management visit count, resource utilization band (an estimate of the intensity of services expected to be needed for the patient), comorbidity status, and probability of being placed under inpatient care. Despite this large number of variables, the predictive ability of the model was smaller than hoped (Nagelkerke R2 = .187, accuracy = 73.1%, Sensitivity = 74.0%, Specificity = 73.1%, Precision = 9.3%).

The primary cause of whether a patient receives CRS services is whether the patient has social needs of which the medical provider is aware. Despite the large number of available variables in the administrative database, these needs were not routinely asked about or entered into administrative data. This is why the predictive ability of the model was small. Therefore, we chose to apply a second stage to the research selection process. In this second stage, we developed an algorithm based on the propensity score model described above, with variables weighted by their coefficients, and applied this algorithm to subsequent data from patients to identify those who were similar to patients who received CRS services. These patients, along with those who received CRS services, were subsequently invited to participate in a baseline survey, which included two items to screen for CRS eligibility (“How easy or difficult is it for you to follow through with your care plan?” and “Which, if any, of the following resources would you like to receive help with at this time”). Patients who responded that it was “difficult” or “very difficult”, and/or patients who endorsed at least one of the 13 resources (e.g. food, housing, transportation) were considered eligible for CRS services and included in the baseline and follow-up surveys. This process produced treatment and comparison groups that were generally balanced across all propensity score variables, although some imbalance remained, which led to the third stage of the propensity score modeling process.

In the third stage, after all baseline and follow-up data were collected, we used propensity score regression adjustment to further balance the groups. We applied multinomial regression to predict whether the patient had one CRS encounter or two or more encounters. All variables from the first round of propensity score modeling were included in this modeling, along with additional variables collected from the baseline measure and which were found to be associated with CRS status: whether the patient specifically had needs for food, housing, transportation, or utilities. We obtained two propensity scores for each individual, which were then included as covariates in mixed effects modeling to analyze group differences in change over time.
