# Supplementary material for: Optimizing the community resource specialist to address social needs in primary care: results from a pragmatic quality improvement evaluation
Source: BMC Prim Care. 2025 Oct 31;26:330. doi: 10.1186/s12875-025-02922-x (PMC12577139; doi:10.1186/s12875-025-02922-x)
Supplement: Supplementary file 3 — Supplementary Material 3. [file 12875_2025_2922_MOESM3_ESM.pdf]

PSC participants only

Thank you for your interest in completing our survey. Before we begin, we have a couple of questions to determine if you are eligible. There are no right or wrong answers, please answer honestly.

A Care Plan is a personalized instruction developed with the primary care team to help manage a health condition or multiple conditions.

How easy or difficult is it for you to follow through with your care plan? For example, is it easy or difficult for you to take your medicine, connect with referrals, follow the exercise recommendations from your doctor, and do the other things that are on your care plan?

a) (1) Very easy
b) (2) Somewhat easy
c) (3) Somewhat difficult
d) (4) Very difficult

Which, if any, of the following would you like to receive help with at this time? *(Select ALL that apply)*

- ☐ Food
- ☐ Housing
- ☐ Transportation
- ☐ Utilities *(heat, electricity, water, etc.)*
- ☐ Medical care, medicine, medical supplies
- ☐ Dental or Vision services
- ☐ Applying for public benefits *(WIC, SSI, SNAP, etc.)*
- ☐ Opportunities for social connection
- ☐ More help with activities of daily living
- ☐ Childcare/other child-related issues
- ☐ Debt (causing financial distress)
- ☐ Legal issues
- ☐ Employment
- ☐ Other
- ☐ I don't want help with any of these

PSC Eligible if:

- Respond c or d on question 1 AND/OR endorse any need on question 2 (any responses other than "I don't want help with any of these")
- If eligible:
  - Phone: Thank you for responding to those questions. You are eligible to complete the full survey. May we begin?
  - Web: Thank you for your responses. You are eligible to complete the full survey (click next to begin)

PSC Ineligible if:

- Respond a or b on question 1 AND respond "I don't want help with any of these" on question 2
- If ineligible
  - Phone: Thank you for responding to those questions. Based on your responses, you are not eligible to complete the full survey. We appreciate your time and willingness to help Kaiser Permanente improve programs for our members.

- Web: Thank you for your responses. Based on your responses, you are not eligible to complete the full survey. We appreciate your time and willingness to help Kaiser Permanente improve programs for our members.

1. On a scale from 1 to 10, where 1 is not at all, and 10 is completely, how much do you trust your health care provider(s) at Kaiser Permanente?

☐ 1 ☐ 2 ☐ 3 ☐ 4 ☐ 5 ☐ 6 ☐ 7 ☐ 8 ☐ 9 ☐ 10

Not at all Completely

A Care Plan is a *personalized instruction developed with the primary care team to help manage a health condition or multiple conditions.*

Q2-5 CRS participants only

2. How easy or difficult is it for you to follow through with your care plan? For example, is it easy or difficult for you to take your medicine, connect with referrals, follow the exercise recommendations from your doctor, and do the other things that are on your care plan?

e) (1) Very easy
f) (2) Somewhat easy
g) (3) Somewhat difficult
h) (4) Very difficult

If phone visit: After a recent visit to a Kaiser Permanente primary care clinic, you spoke with a Community Resource Specialist (CRS named ____) over the phone to talk about some of the issues you may be facing in your life.

If in person visit: During a recent visit to a Kaiser Permanente primary care clinic, you met with a Community Resource Specialist (CRS named ____) to talk about some of the issues you may be facing in your life.

(A community resource specialist is someone from your primary care clinic who may have called you or that you saw in person about some of the issues you may be facing in your life.)

Reflecting on that interaction, please respond how much you agree or disagree with the following statements

3. Meeting with the CRS was worth my time.

(1) Strongly Disagree (2) Disagree (3) Undecided (4) Agree (5) Strongly Agree

4. Meeting with the CRS addressed needs that allowed me to follow my provider's care plan.

(1) Strongly Disagree (2) Disagree (3) Undecided (4) Agree (5) Strongly Agree

5. I feel supported by the CRS.

(1) Strongly Disagree (2) Disagree (3) Undecided (4) Agree (5) Strongly Agree

The next questions are about your experiences with your Care Team.
"Care Team" refers to all of the individuals at the primary care clinic who may have been involved in your recent visit, including physicians, nurses, medical assistants, pharmacists, social workers, or other health care providers. The following are sentences that describe some of the different ways you might have thought or felt about your care team. Please indicate how often, if at all, you have felt that way.

6. **My care team and I agree about the things I will need to do in our work together to help improve my situation.**
   Would you say you felt that way…
   (1) Never (2) Rarely (3) Occasionally (4) Sometimes (5) Often (6) Very Often (7) Always

7. **What I am doing with my care team gives me new ways of looking at my problem.**
   Would you say you felt that way…
   (1) Never (2) Rarely (3) Occasionally (4) Sometimes (5) Often (6) Very Often (7) Always

8. **I believe my care team likes me.**
   Would you say you felt that way…
   (1) Never (2) Rarely (3) Occasionally (4) Sometimes (5) Often (6) Very Often (7) Always

9. **My care team does not understand what I am trying to accomplish in our work together.**
   Would you say you felt that way…
   (1) Never (2) Rarely (3) Occasionally (4) Sometimes (5) Often (6) Very Often (7) Always

10. **I am confident in my care team's ability to help me.**
    Would you say you felt that way…
    (1) Never (2) Rarely (3) Occasionally (4) Sometimes (5) Often (6) Very Often (7) Always

11. **My care team and I are working towards mutually agreed upon goals.**
    Would you say you felt that way…
    (1) Never (2) Rarely (3) Occasionally (4) Sometimes (5) Often (6) Very Often (7) Always

12. **I feel that my care team appreciates me.**
    Would you say you felt that way…
    (1) Never (2) Rarely (3) Occasionally (4) Sometimes (5) Often (6) Very Often (7) Always

13. **We agree on what is important for me to work on.**
    Would you say you felt that way…
    (1) Never (2) Rarely (3) Occasionally (4) Sometimes (5) Often (6) Very Often (7) Always

14. **My care team and I trust one another.**

Would you say you felt that way…
(1) Never (2) Rarely (3) Occasionally (4) Sometimes (5) Often (6) Very Often (7) Always

15. **My care team and I have different ideas on what my problems are.**
    Would you say you felt that way…
    (1) Never (2) Rarely (3) Occasionally (4) Sometimes (5) Often (6) Very Often (7) Always

16. **We have established a good understanding of the kind of changes that would be good for me.**
    Would you say you felt that way…
    (1) Never (2) Rarely (3) Occasionally (4) Sometimes (5) Often (6) Very Often (7) Always

17. **I believe the way we are working with my problem is correct.**
    Would you say you felt that way…
    (1) Never (2) Rarely (3) Occasionally (4) Sometimes (5) Often (6) Very Often (7) Always

For the next set of questions, choose the option that best describes your opinion.

18. **If someone opposes me, I can find the means and ways to get what I want. Would you say that's…**
    (1) not at all true (2) hardly true (3) moderately true (4) exactly true

19. **It is easy for me to stick to my aims and accomplish my goals.**
    (1) not at all true (2) hardly true (3) moderately true (4) exactly true

20. **I am confident that I could deal efficiently with unexpected events.**
    (1) not at all true (2) hardly true (3) moderately true (4) exactly true

21. **Thanks to my resourcefulness, I know how to handle unforeseen situations.**
    (1) not at all true (2) hardly true (3) moderately true (4) exactly true

22. **I can remain calm when facing difficulties because I can rely on my coping abilities.**
    (1) not at all true (2) hardly true (3) moderately true (4) exactly true

23. **I can usually handle whatever comes my way.**
    (1) not at all true (2) hardly true (3) moderately true (4) exactly true

Over the past <u>2 weeks</u>, how often have you been bothered by any of the following problems?

24. **Little interest or pleasure in doing things**
    (0) not at all (1) several days (2) more than half the days (3) nearly every day

25. **Feeling down, depressed or hopeless**
    (0) not at all (1) several days (2) more than half the days (3) nearly every day

26. **Feeling nervous, anxious, or on edge**

(0) not at all (1) several days (2) more than half the days (3) nearly every day

27. Not being able to stop or control worrying
(0) not at all (1) several days (2) more than half the days (3) nearly every day

YOUR CURRENT LIFE SITUATION

Kaiser Permanente is investing in expanding care for the whole person, including social, financial, and basic resource needs. Please answer the following questions to help us better understand you and your current situation so that we can inform our future efforts.

28. Which of the following best describes your current living situation? *(Select ONE only)*

☐ Live alone in my own home (house, apartment, condo, trailer, etc.); may have a pet
☐ Live in a household with other people
☐ Live in a residential facility where meals and household help are routinely provided by paid staff (or could be if requested)
☐ Live in a facility such as a nursing home which provides meals and 24-hour nursing care
☐ Temporarily staying with a relative or friend
☐ Temporarily staying in a shelter or homeless
☐ Other

29. Do you have any concerns about your current living situation, like housing conditions, safety, and costs?

☐ Yes →
☐ No

Which of the following concerns do you have?
☐ Condition of housing ☐ Lack of more permanent housing
☐ Ability to pay for housing or utilities ☐ Feeling safe ☐ Other please describe the concern)

30. In the past *3 months*, did you have trouble paying for any of the following? *(Select ALL that apply, answer yes or no to each if on the phone)*

☐ Food ☐ Housing ☐ Heat and electricity ☐ Medical needs ☐ Transportation
☐ Childcare ☐ Debts ☐ Other ☐ None of these

31. In the past *3 months*, how often have you worried that your food would run out before you had money to buy more?

☐ Never ☐ Sometimes ☐ Often ☐ Very often

32. Are you easily able to get enough healthy food to eat?

☐ Yes ☐ No

33. Has lack of transportation kept you from medical appointments or from doing things needed for daily living? *(Select ALL that apply)*

☐ Kept me from medical appointments or from getting medications
☐ Kept me from doing things needed for daily living
☐ Not a problem for me

34. If for any reason you need help with activities of daily living such as bathing, preparing meals, shopping, managing finances, etc., do you get the help that you need?

☐ I don't need any help ☐ I get all the help I need ☐ I could use a little more help ☐ I need a lot more help

35. How often do you feel lonely or isolated from those around you?

☐ Never ☐ Rarely ☐ Sometimes ☐ Often ☐ Always

36. Which of the following are the biggest problems for you right now?

☐ Food
☐ Housing
☐ Transportation
☐ Utilities *(heat, electricity, water, etc.)*
☐ Medical care, medicine, medical supplies
☐ Dental or Vision services
☐ Applying for public benefits *(WIC, SSI, SNAP, etc.)*
☐ Opportunities for social connection
☐ More help with activities of daily living
☐ Childcare/other child-related issues
☐ Debt (causing financial distress)
☐ Legal issues
☐ Employment
☐ Other
☐ None
☐ I don't want help with any of these

→ 36a. On a scale from 0, not at all) to 10, very much, how much of a problem is this for you?

☐

CRS participants only

37. Which, if any, of the following would you like to receive help with at this time? *(Select ALL that apply)*

☐ Food
☐ Housing
☐ Transportation
☐ Utilities *(heat, electricity, water, etc.)*
☐ Medical care, medicine, medical supplies
☐ Dental or Vision services
☐ Applying for public benefits *(WIC, SSI, SNAP, etc.)*
☐ Opportunities for social connection
☐ More help with activities of daily living
☐ Childcare/other child-related issues
☐ Debt (causing financial distress)
☐ Legal issues
☐ Employment
☐ Other
☐ I don't want help with any of these

The next statements refer to some ways that people may respond when under a lot of stress. Please tell me how <u>often you usually do each</u> of the following when under a lot of stress.

38. I concentrate my efforts on doing something about the situation I'm in.

(1) No, I don't do this at all
(2) I do this a little bit
(3) I do this a medium amount

(4) I do this a lot

39. I get emotional support from others.

(1) No, I don't do this at all
(2) I do this a little bit
(3) I do this a medium amount
(4) I do this a lot

40. I give up trying to deal with it.

(1) No, I don't do this at all
(2) I do this a little bit
(3) I do this a medium amount
(4) I do this a lot

41. I take action to try to make the situation better.

(1) No, I don't do this at all
(2) I do this a little bit
(3) I do this a medium amount
(4) I do this a lot

42. I get help and advice from other people.

(1) No, I don't do this at all
(2) I do this a little bit
(3) I do this a medium amount
(4) I do this a lot

43. I try to see it in a different light, to make it seem more positive.

(1) No, I don't do this at all
(2) I do this a little bit
(3) I do this a medium amount
(4) I do this a lot

44. I try to come up with a strategy about what to do.

(1) No, I don't do this at all
(2) I do this a little bit
(3) I do this a medium amount
(4) I do this a lot

45. I get comfort and understanding from someone.

(1) No, I don't do this at all
(2) I do this a little bit
(3) I do this a medium amount
(4) I do this a lot

46. I give up the attempt to cope.

(1) No, I don't do this at all
(2) I do this a little bit
(3) I do this a medium amount
(4) I do this a lot

47. I look for something good in what is happening.
(1) No, I don't do this at all
(2) I do this a little bit
(3) I do this a medium amount
(4) I do this a lot

48. I try to get advice or help from other people about what to do.
(1) No, I don't do this at all
(2) I do this a little bit
(3) I do this a medium amount
(4) I do this a lot

49. I think hard about what steps to take.
(1) No, I don't do this at all
(2) I do this a little bit
(3) I do this a medium amount
(4) I do this a lot

50. In the past 30 days, for how many days were you totally unable to carry out your usual activities or work because of any health condition?

☐

51. In the past 30 days, not counting the days that you were totally unable, for how many days did you cut back or reduce your usual activities or work because of any health condition?

☐

52. In general, would you say your health is… (Choose ONE only)
(a) Excellent (b) Very good (c) Good (d) Fair (e) Poor

Thank you for completing the survey. We will mail $20 in the next few weeks as a thank you.

We will contact you again for another survey in 3 months.

Would you prefer to do the next survey by phone or online?
